# Comparison of visual quality after EVO-ICL implantation and SMILE to select the appropriate surgical method for high myopia

**DOI:** 10.1186/s12886-019-1029-x

**Published:** 2019-02-08

**Authors:** Qin Qin, Lianyun Bao, Liping Yang, Zifang He, Zhenping Huang

**Affiliations:** 1 Department of Ophthalmology, Nanjing Drum Tower Hospital Clinical College of Nanjing Medical University, Nanjing, 210008 Jiangsu China; 2 Department of Ophthalmology, Nanjing Drum Tower Hospital, Affiliated to Nanjing University Medical School, Nanjing, 210008 China; 3 Department of Ophthalmology, Jinling Medical School of Nanjing Medical University, No. 305 East Zhongshan Road, Nanjing, 210002 Jiangsu Province China; 4 Department of Ophthalmology, Jinling Hospital, No. 305 East Zhongshan Road, Nanjing, 210002 Jiangsu Province China

**Keywords:** Myopia, EVO intraocular collamer lens, Small-incision lenticule extraction

## Abstract

**Background:**

This study sought to compare the visual quality between intraocular collamer lens (EVO-ICL) implantation and small-incision lenticule extraction (SMILE) and determine the appropriate surgical method to treat patients with high myopia (− 6.25 to − 10 D).

**Methods:**

A total of 48 eyes underwent EVO-ICL implantation and another 48 eyes underwent SMILE. The uncorrected distance visual acuity (UDVA), best-corrected distance visual acuity (BCVA) and equivalent spherical degree were compared across the SMILE (− 6.25 to − 10 D) and EVO-ICL (− 6.25 to − 10 D) implantation groups. Preoperative and postoperative visual quality parameters were compared between and within groups.

**Results:**

The OQAS II values (OV 100%) one week and one month after surgery and the modulation transfer function (MTF), OV 20% and OV 9% values one week after surgery in the SMILE group were lower than the respective preoperative values. The objective scatter index (OSI) value increased one week as well as one month after surgery compared with the preoperative values. The MTF cut-off value of the SMILE group was lower than that of the EVO-ICL implantation group three months after surgery.

**Conclusions:**

For patients with high myopia, the postoperative visual quality of EVO- ICL implantation was slightly better than that of SMILE.

## Background

Myopia refers to a situation in which the light through eyes is focused in front of the retina [1]. Due to the increasing high prevalence over the past few decades, myopia remains a significant public health issue in some areas of the world, especially East Asia [2]. Myopia is measured in dioptres and divided into four status groups (low, moderate, high and severe) based on the pathogenesis [3, 4]. Although treatments including implantable collamer lens (ICL) and small-incision lenticule extraction (SMILE) have been widely used to correct near-sightedness, the optimal surgical methods for patients with high myopia still remains controversial.

The Visian ICL™ (STAAR Surgical, Nidau, Switzerland) is a posterior chamber phakic intraocular lens (IOL) [5–7]. Recently, a novel ICL operation based on an artificial hole (EVO Visian Implantable Collamer Lens) has been developed. Previous work shows that EVO-ICL implantation is satisfactory in terms of safety [8]. Moreover, EVO-ICL implantation is similar to the traditional ICL implantation with regard to inducing the higher-order aberrations and contrast sensitivity function [9]. Furthermore, the laser-assisted in situ keratomileusis (LASIK), major myopia corneal laser-assisted surgery, has been available for more than 30 years [10]. LASIK is accepted by both doctors and patients due to the accuracy and fast recovery in patients [11]. SMILE, also known as the flapless surgical approach, does not necessitate the lifting of the corneal flap. Because the risk of flap-related complications are reduced, SMILE may therefore have advantages over LASIK [12]. Furthermore, the early clinical outcomes of SMILE to correct myopia and myopic astigmatism are encouraging [13–18]. Even though the parameters for optical quality as well as intraocular scattering is valuable for the subsequent satisfaction and postoperative visual performance in myopia, the comparison between patients with SMILE and EVO-ICL implantation of these parameters is still unclear.

**Fig. 1:**
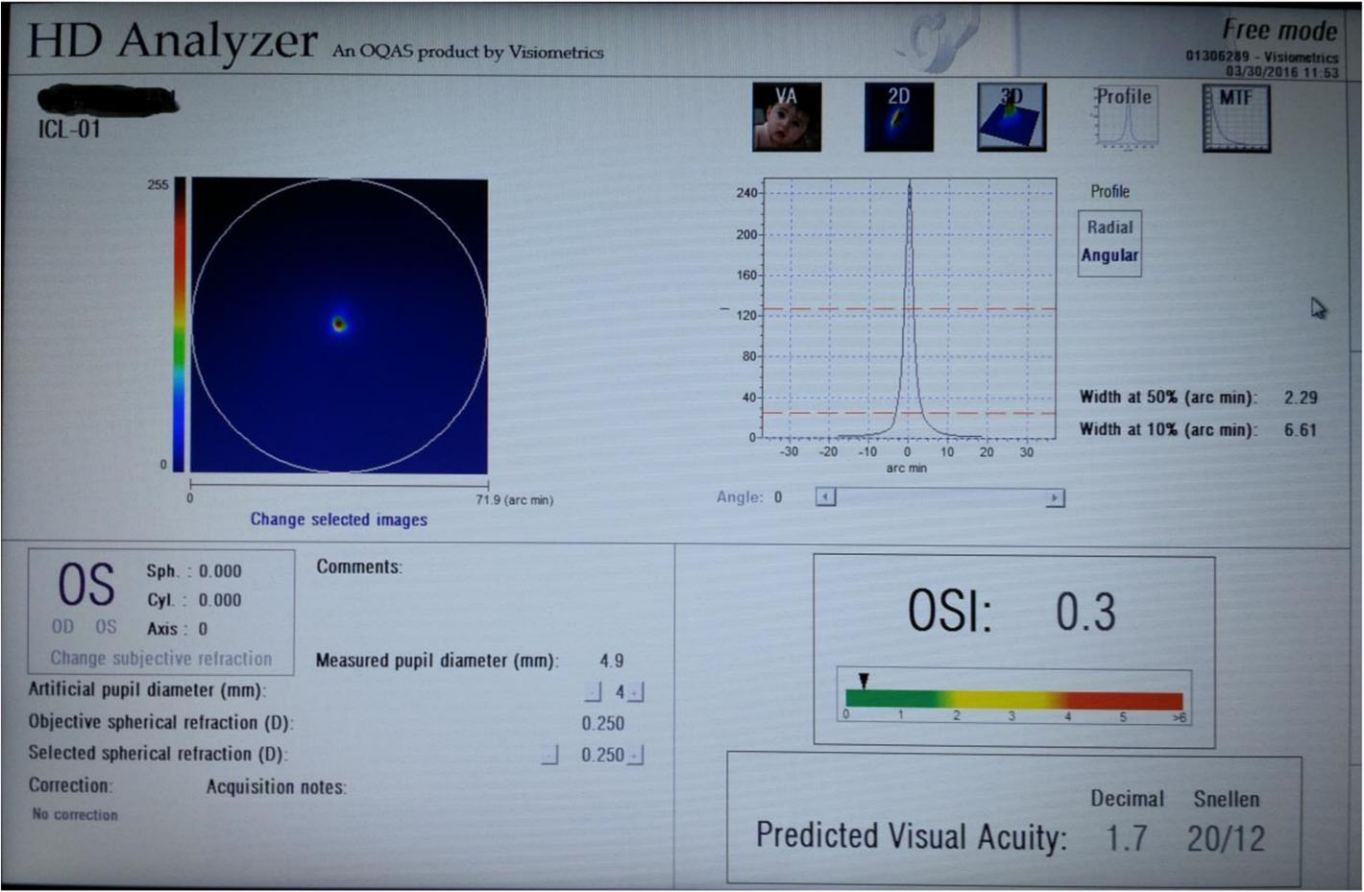
OQAS II detection figure

In the present study, the double-pass technique (via OQAS™ II, Optical Analysis System, Visiometrics, Spain) was used to assess optical quality parameters and intraocular scattering in patients who had undergone EVO-ICL implantation or SMILE (Fig. 1). This comparative clinical study assessed the application scope of two types of surgical methods to treat high myopia. The findings of this study might help us to select the appropriate surgical methods for patients with high myopia.

## Methods

### Patients

In the first group, 48 eyes from 24 consecutive patients (13 women, 11 men; aged 20–34 years) who underwent the bilateral implantation of the posterior chamber phakic ICL with a 0.36 mm central artificial hole (EVO-ICL™, STAAR Surgical) of myopia (manifest refraction spherical equivalent to − 6.25 to − 10.0 dioptres [D], manifest cylinder < 0.5 D, chamber depth ≥ 3.0 mm, endothelial cells ≥2500/mm^2^) were assessed. The D stability of these patients was more than two years (an increase of <− 0.5 D every year). Meanwhile, 48 eyes from 24 age-matched patients (12 women, 12 men; aged 20–31 years) who underwent bilateral SMILE to correct myopia and myopic astigmatism (manifest refraction spherical equivalent of − 6.25 to − 10 D, manifest cylinder of 0 to 1.00 D) of sufficient corneal thickness (estimated residual thickness of the stromal bed > 250 μm) at the Jinling Clinical Medical College of Nanjing Medical University and Nanjing Drum Tower Hospital Clinical College of Nanjing Medical University were enrolled in the current study. Patients with a history of ocular surgery, severe dry eye, progressive corneal degeneration, cataract or uveitis were excluded. Based on OCULYZERII (WaveLight, Alcon, Fort Worth, TX, USA), eyes with keratoconus were excluded because early keratoconus without clinical features could increase the security of corneal refractive surgery and prevent the occurrence of postoperative ectasia [19, 20]. The institutional review board at Nanjing Medical University approved this study, which followed the tenets of the Declaration of Helsinki. Written informed consent was obtained from all patients after the nature and possible consequences of the study were explained.

### Operation procedure and follow-up period

Before surgery, non-cycloplegic autorefraction, corneal topography, the uncorrected visual acuity (UCVA), the best-corrected visual acuity (BCVA), cycloplegic refraction, intraocular pressure, axial length, visual quality and scotopic pupil size were checked. Next, three mirror contact lenses were used to examine the retina. The laser photocoagulation treatment was administered for severe retinal degeneration or a torn hole. Anterior chamber depth, white-to-white distance, ciliary sulcus spacing and corneal endothelial cell counts were measured in the EVO-ICL implantation group. Antibiotic eye drops were used three times a day preoperatively. In this study, the same doctor was in charge of all surgeries.

### SMILE group

After the surface anaesthesia with 0.4% oxybuprocaine, the VisuMax femtosecond laser system (Carl Zeiss, Meditec AG, Germany, 500-kHz repetition rate) was performed on SMILE. A small (S) curved interface cone was used during each surgery. The anterior surface of the lenticule (spiral-out pattern) and the posterior surface of the lenticule (spiral-in pattern) were followed by a side cut of the cap. The power and spot distances for lenticule creation were 140 nJ power and 3.0 μm, respectively. Parameters for the femtosecond laser were 6.5 mm lenticule diameter, 120 μm cap thickness, a 3-mm side cut for the access to the lenticule with angles of 90°7.5 mm cap diameter and spot distance 2.0 μm for a side cut. A spatula was inserted through the side cut over the roof of the refractive lenticule to dissect this plane, followed by the bottom of the lenticule. The lenticule was subsequently grasped with modified McPherson forceps (Geuder, GmbH, Heidelberg, Germany) and removed. After the removal of the lenticule, the intrastromal space was flushed with a balanced salt solution. After surgery, eye drops of tobramycin-dexamethasone (Tobradex, Alcon, Fort Worth, TX, USA) were administered into the eyes; this intervention was performed four times every day for one week. Flumetholon (0.1% fluorometholone; Santen, Japan) was used four times a day for the second week; after which the frequency decreased by one administration per day each week for one month. Finally, 0.5% antibiotic (levofloxacin; Santen, Japan) was administered topically four times every day for two weeks.

### EVO-ICL group

The manufacturer performed the ICL power calculation (STAAR Surgical) by the modified vertex formula based on the ICL Power Calculation Software (http://en.informer.com/icl-power-calculation-software/, version 3.0). To decrease the preoperative refractive errors in each patient’s eye, the target refraction was based on emmetropia. The panoramic ultrasound biomicroscope (UBM), which used to the study of anterior segment structures of human eye, could measure central corneal thickness (CCT), central anterior chamber depth (CACD) and anterior chamber angle etc. [21]. The manufacturer also decided the ICL size according to the parameter of horizontal corneal diameter, which was measured with a vernier caliper and the sulcus-to-sulcus (STS) distance using a panoramic UBM. We calculated the ideal ICL size as STS + 0.7 mm. The anterior chamber depth and corneal curvature were measured with OCULYZERII (WaveLight, Alcon).

On the day of their surgery, patients were administered dilating and cycloplegic agents. After peribulbar anaesthesia, an EVO-ICL was inserted through a 3 mm clear corneal incision by the injector cartridge (STAAR Surgical) after the placement of hyaluronate (Shandong Bausch & Lomb Freda Company) into the anterior chamber. After surgery, steroidal (1% prednisolone acetate; Allergan, Ireland) and a 0.5% antibiotic (levofloxacin; Santen, Japan) was given four times every day for two weeks, after which the dose was decreased gradually.

### Measurement of visual quality

After surgery, the optical quality and objective intraocular scattering measurements were performed with OQAS™ II (Optical Analysis System, Visiometrics, Spain) in a dark environment (approximately 25 lx) preoperatively and at one week, one month and three months (for a 4.0 mm pupil). The device (OQAS II) has acceptable reliability, and the eye’s realignment does not alter the measurements [22, 23]. The double-pass technique enables the assessment of the retinal image quality only with one specific pupil diameter per measurement; an additional measurement is required for other desired pupil sizes. Therefore, retinal image quality measures were assessed with a 4.0 mm pupil in this study. This standard size is often used to analyse ocular aberrations and it more closely simulates visual acuity measurements performed with an undilated pupil [24].

The instrument automatically corrected spherical ametropia between + 6 and − 8 D. Ametropia beyond the spherical range or higher than the 0.5 D cylinder required an additional lens on the instrument insert frame. The patient blinked a few times before each inspection to spread evenly over the tear film. The instrument was based on the double-pass technique to directly obtain a point-source retinal image analysis, and then the point spread function (PSF) was analysed. The objective visual analysis system of OQAS II™ is based on the double-pass retinal imaging technique used in this study. The distribution of light intensity in the retina of the 780 nm infrared acquisition point-source imaging, with a description of the point-source resolution of the PSF of optical system expression analysis of intraocular optical imaging quality, and modulation transfer function was obtained by PSF. The optical quality and objective intraocular scattering parameters were analysed by PSF, including cut-off frequency of the modulation transfer function (MTF cut-off), Strehl ratio (SR), OQAS under different contrast value (OQAS values (OV) 100, 20, 9%) and the objective scatter index (OSI). OQAS values under 100, 20 and 9% contrast ratio are below the MTF cut-off frequency, and the 0.05 and 0.01 MTF values are segmented by 30C/deg., respectively.

### Statistical analysis

SPSS 20.0 was used as the statistical software in the current study. UDVA, BCVA, spherical equivalent, optical quality, scattering function results, comparison of corneal endothelial cell density were compared between two groups. These parameters were also compared pre- and postoperatively within each group using generalised estimating equations tests. A *p*-value of <0.05 was considered significant.

## Results

### Follow-up and baseline comparison

No patients in this study were lost prior to the three-month follow-up. Our study found that differences in preoperative equivalent spherical D, UDVA and BCVA between the SMILE (− 6.25 to − 10.0 D) and EVO-ICL (− 6.25 to − 10.0 D) groups were not significant (Table 1). None of the patients had obvious complications during either surgery. Some patients in the SMILE group exhibited transient haze during the early postoperative period. Ocular pain and corneal oedema were observed in two eyes with high intraocular pressure after EVO-ICL implantation. However, the symptoms disappeared 24 h after intravenous infusion of mannitol. The intraocular pressure in another patient was 28–31 mmHg (1 mmHg = 0.133 kPa) two weeks after surgery. Carteolol hydrochloride eye drops were administered twice per day, and the intraocular pressure recovered five days later (17 mmHg). One month after surgery, the intraocular pressure was stable. In addition, none of the patients in the two groups exhibited serious complications, such as epithelial implantation, infectious keratitis, corneal ectasia, endophthalmitis or lens opacity.

**Table 1:** Preoperative demographics of the eyes undergoing SMILE and EVO-ICL

	SMILE (− 6.25D~ − 10D)	EVO-ICL (− 6.25D~ − 10D)	*P* value
Age(years)	23 years (20, 31 years)	24 years (20, 34 years)	
Gender	Male: Female = 12:12	Male:Female = 11:13	
Spherical	−8.00 ± 1.65	−8.15 ± 1.71	*P* = 0.689
Equivalent (D)	(−6.25~ − 9.25)	(− 6.25~ − 9.50)	
LogMAR	1.33 ± 0.23(1.9~0.9)	1.36 ± 0.27(1.9~0.9)	*P* = 0.650
UDVA			
LogMAR	−0.04 ± 0.04(− 0.1~0)	− 0.04 ± 0.05(− 0.1~0)	*P* = 0.763
CDVA			

*P* = *P* value of the data statistically compared between SMILE (−6.25 to − 10 D) group and EVO-ICL implatation (− 6.25 to − 10 D) groups

*LogMAR* logarithm of the minimal angle of resolution, *UDVA* uncorrected distance visual acuity, *CDVA* corrected distance visual acuity, *D* diopter

### Safety and efficacy comparison between different groups

All procedures showed acceptable safety and efficacy after surgery, and the patients did not experience BCVA loss. The safety indexes of the SMILE group and the EVO-ICL implantation group were 1.00 ± 0.01 and 1.01 ± 0.02, respectively. No significant difference was found between these two groups. The efficacy index of the SMILE group was 1.00 ± 0.01 and the EVO-ICL implantation group was 1.01 ± 0.01. Again, no significant difference was found between these two groups.

The postoperative visual acuity and refractive power among the different groups are shown in Table 2. First, a comparison within groups was performed: the UDVA in the SMILE and EVO-ICL implantation groups three months after surgery increased compared to preoperative UDVA values, and the difference was significant (*P* < 0.05). No significant difference was found between the preoperative and postoperative BCVA in the SMILE group (*P* = 0.318). The three-month postoperative BCVA values of the EVO-ICL implantation groups were higher than the preoperative values, and these differences were significant (*P* = 0.003). The equivalent spherical Ds three months after surgery in the two groups were lower than their respective preoperative values, and these differences were significant (*P* = 0.000).

**Table 2:** Time courses of the visual and refractive outcomes between the SMILE (− 6.25 to − 10 D) and EVO-ICL (− 6.25 to − 10 D) groups

	Preoperative	Postoperative period			*P*-value
		1 week	1 month	3 month	
	LogMAR UDVA				
SMILE (− 6.25 to − 10 D)	1.33 ± 0.23	−0.01 ± 0.06	− 0.04 ± 0.06	− 0.04 ± 0.07	*P*_0_ = 0.000*
EVO-ICL (− 6.25 to − 10 D)	1.36 ± 0.27	0.00 ± 0.10	−0.04 ± 0.06	− 0.06 ± 0.05	*P*_0_ = 0.000*
*P*-value	*P*_1_ = 0.650	*P*_1_ = 0.448	*P*_1_ = 0.720	*P*_1_ = 0.207	
	LogMAR BCVA				
SMILE (−6.25 to −10 D)	−0.04 ± 0.04	− 0.02 ± 0.06	−0.05 ± 0.05	− 0.05 ± 0.06	*P*_0_ = 0.318
EVO-ICL (− 6.25 to − 10 D)	−0.04 ± 0.05	− 0.04 ± 0.06	−0.05 ± 0.05	− 0.06 ± 0.04	*P*_0_ = 0.003*
*P*-value	*P*_1_ = 0.763	*P*_1_ = 0.324	*P*_1_ = 0.698	*P*_1_ = 0.345	
	spherical equivalent(D)				
SMILE (−6.25 to −10 D)	− 6.00 ± 1.68	0.04 ± 0.19	0.00 ± 0.21	−0.06 ± 0.31	*P*_0_ = 0.000*
EVO-ICL (−6.25 to −10 D)	− 6.15 ± 1.72	0.03 ± 0.20	−0.01 ± 0.20	0.01 ± 0.20	*P*_0_ = 0.000*
P-value	*P*_1_ = 0.189	*P*_1_ = 0.773	*P*_1_ = 0.892	*P*_1_ = 0.285	

*P*_0_ = *P*-value of the difference between the preoperative and three month values; *P*_1_ = *P*-value of the difference between the SMILE (−6.25 to −10 D) and EVO-ICL implantation (− 6.25 to − 10 D) groups; *P < 0.05 = significant difference

*LogMAR* logarithm of the minimal angle of resolution, *UDVA* uncorrected distance visual acuity BCVA best-corrected distance visual acuity, *D* diopter

The difference between UDVA, BCVA or the equivalent spherical D values before the surgery or at any of the postoperative time periods between the SMILE and EVO-ICL implantation groups was not significant.

### Visual quality comparison before and after surgery in the SMILE and EVO-ICL groups

The visual quality in the SMILE group before surgery as well as one week, one month and three months after surgery are listed in Table 3. Besides MTF at one week after surgery, no significant difference was found in the cut-off frequency or the SR in the SMILE group before surgery or at any time point after surgery. The OV 100% values one week and one month after surgery and the MTF, OV 20% and OV 9% values one week after surgery were lower than their respective preoperative values (*P* = 0.040, 0.048, 0.011, 0.002 and 0.001, respectively). The differences in OQAS values before surgery or at three months after surgery were not significant. After surgery, the OSI values at one week as well as one month were significantly higher than those before surgery (*P* = 0.000 and 0.013, respectively), and no significant difference was found in the OSI values before surgery and three months after surgery.

**Table 3:** Time courses of the optical quality parameters after SMILE

	Preoperative	Postoperative period		
		1 week	1 month	3 month
MTF cutoff frequency (cpd)	48.36 ± 5.67	44.81 ± 7.33	46.02 ± 6.06	46.34 ± 4.23
P-value		*P*_1_ = 0.011*	*P*_2_ = 0.126	*P*_3_ = 0.075
SR	0.25 ± 0.05	0.23 ± 0.03	0.25 ± 0.06	0.26 ± 0.06
P-value		*P*_1_ = 0.916	*P*_2_ = 0.947	*P*_3_ = 0.953
OV 100%	1.62 ± 0.14	1.45 ± 0.33	1.48 ± 0.19	1.54 ± 0.16
P-value		*P*_1_ = 0.040*	*P*_2_ = 0.048*	*P*_3_ = 0.130
OV 20%	1.26 ± 0.23	1.10 ± 0.19	1.12 ± 0.03	1.13 ± 0.26
P-value		*P*_1_ = 0.002*	*P*_2_ = 0.062	*P*_3_ = 0.085
OV 9%	0.71 ± 0.14	0.60 ± 0.09	0.65 ± 0.19	0.67 ± 0.13
P-value		*P*_1_ = 0.001*	*P*_2_ = 0.553	*P*_3_ = 0.936
OSI	0.40 ± 0.11	0.55 ± 0.22	0.50 ± 0.18	0.42 ± 0.13
P-value		*P*_1_ = 0.000*	*P*_2_ *=* 0.013*	*P*_3_ = 0.963

*P*_1_ = *P*-value of the difference in the visual quality parameters before surgery and one week after surgery; *P*_2_ = *P*-value of the difference in the visual quality parameters before surgery and one month after surgery; *P*_3_ = P-value of the difference in the visual quality parameters before surgery and three months after surgery

*MTF* modulation transfer function, *OSI* objective scattering index, *OV* OQAS value

*P < 0.05 = significant difference

The comparison of visual quality in the EVO-ICL implantation group before surgery as well as one week, one month and three months after surgery are listed in Table 4. The MTF, SR, OV 100%, OV 20% and OV 9% values one week after surgery were lower than their respective preoperative values (*P* = 0.000, 0.010, 0.002, 0.000 and 0.001, respectively). The OSI values one week after surgery were higher than their respective preoperative values (*P* = 0.003). The difference in the OQAS values before surgery or one to three months after surgery were not significant.

**Table 4:** Time courses of the optical quality parameters after EVO-ICL

	Preoperative	Postoperative period		
		1 week	1 month	3 month
MTF cutoff frequency (cpd)	48.46 ± 5.18	44.48 ± 5.51	47.17 ± 10.23	48.96 ± 3.69
*P*-value		*P*_1_ *=* 0.000*	*P*_2_ = 1.000	*P*_3_ = 1.000
SR	0.26 ± 0.06	0.22 ± 0.03	0.24 ± 0.08	0.26 ± 0.06
*P*-value		*P*_1_ *=* 0.010*	*P*_2_ *=* 0.749	*P*_3_ = 1.000
OV 100%	1.61 ± 0.13	1.45 ± 0.29	1.55 ± 0.33	1.59 ± 0.17
P-value		*P*_1_ *=* 0.002*	*P*_2_ *=* 0.439	*P*_3_ *=* 1.000
OV 20%	1.22 ± 0.21	1.03 ± 0.12	0.12 ± 0.31	1.14 ± 0.25
P-value		*P*_1_ = 0.000*	*P*_2_ = 0.621	*P*_3_ = 0.778
OV 9%	0.72 ± 0.15	0.61 ± 0.09	0.67 ± 0.14	0.69 ± 0.16
*P*-value		*P*_1_ = 0.001*	*P*_2_ = 0.772	*P*_3_ = 1.000
OSI	0.41 ± 0.11	0.54 ± 0.26	0.40 ± 0.55	0.43 ± 0.13
P-value		*P*_1_ = 0.003*	*P*_2_ = 0.633	*P*_3_ = 1.000

*P*_1_ = *P*-value of the difference between visual quality parameters before surgery and one week after surgery; *P*_2_ = *P*-value of the difference between visual quality parameters before surgery and one month after surgery; *P*_3_ = *P*-value of the difference between visual quality parameters before surgery and three months after surgery

*MTF* modulation transfer function, *OSI* objective scattering index, *OV* OQAS value

*P < 0.05 = significant difference

### Visual quality comparison between two groups

Table 5 compares the visual quality of patients with high myopia who received the two types of surgical methods. The visual quality of the SMILE and EVO-ICL groups was compared before surgery as well as one week and three months after surgery. No significant differences were found in the MTF cut-off frequency, SR, OV 100%, OV 20%, OV 9% or OSI between the two groups at any time point. However, the MTF cut-off value in the SMILE group was lower than that in the EVO-ICL implantation group three months after surgery (*P* = 0.049).

**Table 5:** Time courses of the optical quality parameters in the SMILE (−6.25 to −10 D) and EVO-ICL (−6.25 to −10 D) groups

MTF	Preoperative			1 week postoperation			3 month postoperation		
	SMILE		EVO-ICL	SMILE		EVO-ICL	SMILE		EVO-ICL
Cut-off frequency	48.36 ± 5.67		48.46 ± 5.18	44.81 ± 7.33		44.48 ± 5.51	46.34 ± 4.23		48.96 ± 3.69
*P*-value		*P* = 1.000			*P* = 1.000			*P* = 0.049*	
SR	0.25 ± 0.05		0.26 ± 0.06	0.23 ± 0.03		0.22 ± 0.03	0.26 ± 0.06		0.26 ± 0.06
P-value		*P* = 0.999			*P* = 0.590			*P* = 1.000	
OV 100%	1.62 ± 0.14		1.61 ± 0.13	1.45 ± 0.33		1.45 ± 0.29	1.54 ± 0.16		1.59 ± 0.17
P-value		*P* = 1.000			*P* = 1.000			*P* = 0.887	
OV 20%	1.26 ± 0.23		1.22 ± 0.21	1.10 ± 0.19		1.03 ± 0.12	1.13 ± 0.26		1.14 ± 0.25
*P*-value		*P* = 1.000			*P* = 0.359			*P* = 1.000	
OV 9%	0.71 ± 0.14		0.72 ± 0.15	0.60 ± 0.09		0.61 ± 0.09	0.67 ± 0.13		0.69 ± 0.16
P-value		*P* = 1.000			*P* = 1.000			*P* = 1.000	
OSI	0.40 ± 0.11		0.41 ± 0.11	0.55 ± 0.22		0.54 ± 0.26	0.42 ± 0.13		0.43 ± 0.13
*P*-value		*P* = 1.000			*P* = 1.000			*P* = 1.000	

*P*=*P-*value of the difference between visual quality parameters in the SMILE and EVO-ICL implantation groups

*MTF* modulation transfer function, *OSI* objective scattering index, *OV* OQAS value

*P < 0.05 = significant difference

### Comparison of corneal endothelial cell density before and after surgery within and between the SMILE and EVO-ICL groups

Table 6 displays that there was no significant difference in corneal endothelial cell density (ECD) of SMILE (− 6.25 to − 10.0 D) and EVO-ICL (− 6.25 to − 10.0 D) groups before and three months after surgery (*P* > 0.05). There was also no significant difference in the two time points between two groups (*P* > 0.05).

**Table 6:** Comparison of corneal endothelial cell density within and between the SMILE(−6.25 to −10 D) and EVO-ICL (−6.25 to −10 D) groups

	Preoperative	3 month Postoperative
SMILE	2875.88 ± 99.82/ mm^2^	2857.90 ± 94.95/ mm^2^
EVO-ICL	2876.17 ± 102.49/ mm^2^	2836.19 ± 106.37/ mm^2^

Comparisons of ECD within and between the SMILE and EVO-ICL groups were all *P* > 0.05

## Discussion

The most common evaluation methods of visual quality after refractive surgery are subjective measurements of light and shade perception, environmental and contrast visual acuity as well as objective measurements of whole eye and corneal wavefront aberration [25–27]. However, scattering and diffraction are important factors that affect visual quality in humans. Femtosecond stromal interface quality was improved with a lower pulse energy and narrower spot separations than those currently used in clinical settings [28]. Kamiya [29] found that the remaining tissue islands in SMILE patients require mechanical dissection; results also indicated that the cavitation bubbles merge together and impair the subsequent laser beam, which results in an increase in surface irregularity. The results in this study showed that the visual quality of the SMILE group one week after surgery was lower than that before surgery. Actually, the femtosecond lenticule extraction led to a transient decrease in optical quality as well as an enhancement in the intraocular scattering during the early period of postoperative because of mild interface haze formation. The visual performance postoperatively after SMILE has been described as a certain extent due to the higher-order aberrations and visual acuity [12, 18, 30, 31]. In addition, the recovery of visual acuity after SMILE is slightly slower than recovery after other techniques of keratorefractive surgery during the early postoperative period [32, 33]. Kamiya [34] assumed that this delay is caused by corneal scattering rather than higher-order aberrations resulting from surface irregularities, and that this is vital for visual performance after FLEx and SMILE. Similar to the previous study, our results indicated that OSI significantly increased one week after surgery and MTF, OV 100, 20% and OV 9% values decreased one week after SMILE. Therefore, we speculated that the decrease in visual quality one week after SMILE was related to the increase in corneal OSI and higher-order aberrations in corneal morphology changes and transient haze. Patients’ visual quality was improved one month after surgery and was significantly improved three months after surgery. BCVA did not significantly differ before or after surgery perhaps because the haze regression led to improved visual quality.

A previous study [35] showed that compared with wavefront-guided LASIK, the ICL implantation has significantly fewer ocular higher-order aberrations not only in patients with high disease status but also in moderate or low disease status [36]. Buhren et al. [37] also reported that after implantation based on the artisan phakic IOL, the number of higher-order aberrations increased. The patients of the EVO-ICL implantation group in this study had less astigmatism; thus, the postoperative UDVA was less affected. The postoperative UDVA was better than the preoperative BCVA, and the MTF, SR,OV 100%, OV 20% and OV 9% values one week after the surgery were lower than their respective preoperative values. However, OSI values one week after surgery were higher than their respective preoperative values. The differences in the OQAS values before surgery or one and three months after surgery were not significant. We believe that the decrease of visual quality one week after surgery may be related to the early postoperative inflammatory response. To optimise ICL implantation visual performance, parameters such as increased higher-order aberrations and decreased retinal magnification might be useful and valuable [35, 36, 38–41]. In the present study, after ICL implantation, the excellent optical quality was in accordance with previous studies. Previous studies have suggested that the implantation of ICL increases the amount of intraocular refractive medium, which might lead to more intraocular scattering. Nevertheless, we hypothesise that ICL would not produce more intraocular scattering because the thickness of the EVO-ICL loop is 100–200 μm, the optical zone thickness is only 50–60 μm and the ICL is located in the ciliary sulcus, which rarely tilts or shifts. Even the visual quality of patients with severe myopia and unhealthy fundus was partially improved.

With the increase in myopia, night glare and blurred vision, the visual quality problems caused by corneal aspheric changes increased spherical aberration and other higher-order aberrations after SMILE in patients with high myopia [12, 14, 15, 31]. In addition, the corneal wound healing response, refractive regression [42] and the security problem make corneal refractive surgery a less attractive choice for patients with high myopia. However, patients with EVO-ICL implantation retained normal corneal morphology to avoid increasing cornea scattering and the increase in OSI and higher-order aberrations was lower. In the present study, the visual quality of the EVO-ICL implantation group and the SMILE group with high myopia did not show a significant difference except for the MTF value at three months after surgery. The results showed that SMILE and EVO-ICL might both achieve satisfactory, safe and effective postoperative visual quality in patients with high myopia. After the posterior chamber IOL implantation in the high myopia patients, the imaging of the external object in the retina was almost the same as that of emmetropic eyes. The retinal magnification of corneal surgery was 0.97, which was less than that of the posterior chamber IOL, which was close to that of the eye node, with a magnification of 1.0. Moreover, our results showed that there was no significant difference in corneal endothelial cell density (ECD) between and within the two groups before and three months after surgery. We speculated that the visual quality in patients with high myopia after EVO-ICL implantation was slightly better than SMILE three months after surgery. There were some limitations in this study. The optical parameters were assessed only for a 4.0 mm pupil. Additional research is necessary for confirmation of optical quality parameters under natural viewing conditions before and after surgery.

## Conclusions

In conclusion, the postoperative visual quality after EVO-ICL implantation was a little better than that after SMILE in patients with − 6.25 to − 10.0 D myopia. If the corneal thickness is limited, patients with larger refractive regression may be expected. Therefore, EVO-ICL implantation is a better first choice. Meanwhile, the appropriate surgical procedure should be chosen based on the preoperative examination, which includes the thickness of the cornea, ACD, ECD etc. and the patient’s own needs, such as financial status.

## Abbreviations

BCVA: Best-corrected distance visual acuity
ICL: Intraocular collamer lens
MTF: Modulation transfer function
OSI: Objective scatter index
OV: OQAS II values
UDVA: Uncorrected distance visual acuity
